# Supplementary material for: Biocatalytic Preparation of Chloroindanol Derivatives. Antifungal Activity and Detoxification by the Phytopathogenic Fungus Botrytis cinerea
Source: Plants (Basel). 2020 Nov 25;9(12):1648. doi: 10.3390/plants9121648 (PMC7759767; doi:10.3390/plants9121648)
Supplement: Supplementary file 1 [file plants-09-01648-s001.pdf]

Supplementary materials

Biocatalytic Preparation of Chloroindanol Derivatives. Antifungal Activity and Detoxification by the Phytopathogenic Fungus *Botrytis cinerea*

Cristina Pinedo-Rivilla [1], Javier Moraga [1,2], Guillermo Pérez-Sasián [1], Alba Peña-Hernández [1], Isidro G. Collado [1] and Josefina Aleu [1,*]

[1] Departamento de Química Orgánica, Facultad de Ciencias, Universidad de Cádiz, 11510 Puerto Real, Cádiz, Spain; cristina.pinedo@uca.es (C.P.-R.); javier.moraga@uca.es (J.M.); guille.perezsasian@gmail.com (G.P.-S.); albaph93@gmail.com (A.P.-H.); isidro.gonzalez@uca.es (I.G.C.)

[2] Departamento de Biomedicina, Biotecnología y Salud Pública, Área de Microbiología, Facultad de Ciencias, Universidad de Cádiz, 11510 Puerto Real, Cádiz, Spain

* Correspondence: josefina.aleu@uca.es; Tel.: +34-956-012747

TABLE OF CONTENTS

Figure S1. ^{1}H NMR spectrum of *anti*-(+)-**7** in $CDCl_3$…..……….....................................S3
Figure S2. ^{13}C NMR spectrum of *anti*-(+)-**7** in $CDCl_3$……………………….…..……….....S4
Figure S3. ^{1}H NMR spectrum of *anti*-(+)-**8** in $CDCl_3$……...S5
Figure S4. ^{13}C NMR spectrum of *anti*-(+)-**8** in $CDCl_3$……………………….…..............S6
Figure S5. ^{1}H NMR spectrum of *syn*-(-)-**8** in $CDCl_3$……………………...……………..S7
Figure S6. ^{13}C NMR spectrum of *syn*-(-)-**8** in $CDCl_3$…….……………………………..…..S8
Figure S7. ^{1}H NMR spectrum of *anti*-(+)-**9** in $CDCl_3$……………………………............S9
Figure S8. ^{13}C NMR spectrum of *anti*-(+)-**9** in $CDCl_3$……….…………………………....S10
Figure S9. Tables of fungal growth inhibition for *Botrytis cinerea* UCA992……...…....S11

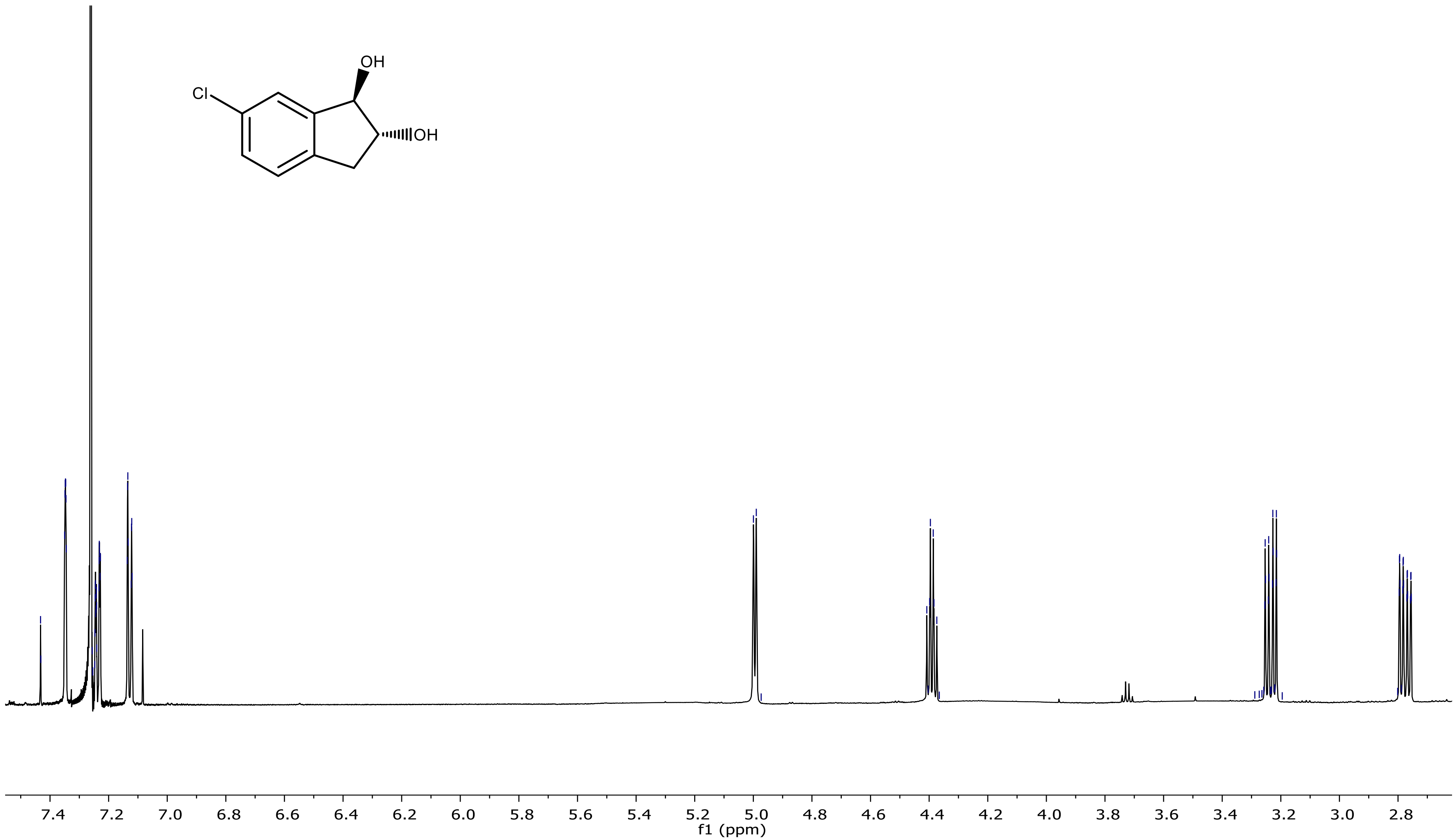

Figure S1. ^{1}H NMR spectrum of *anti*-(+)-6-chloroindan-1,2-diol (*anti*-(+)-**7**) in $CDCl_3$.

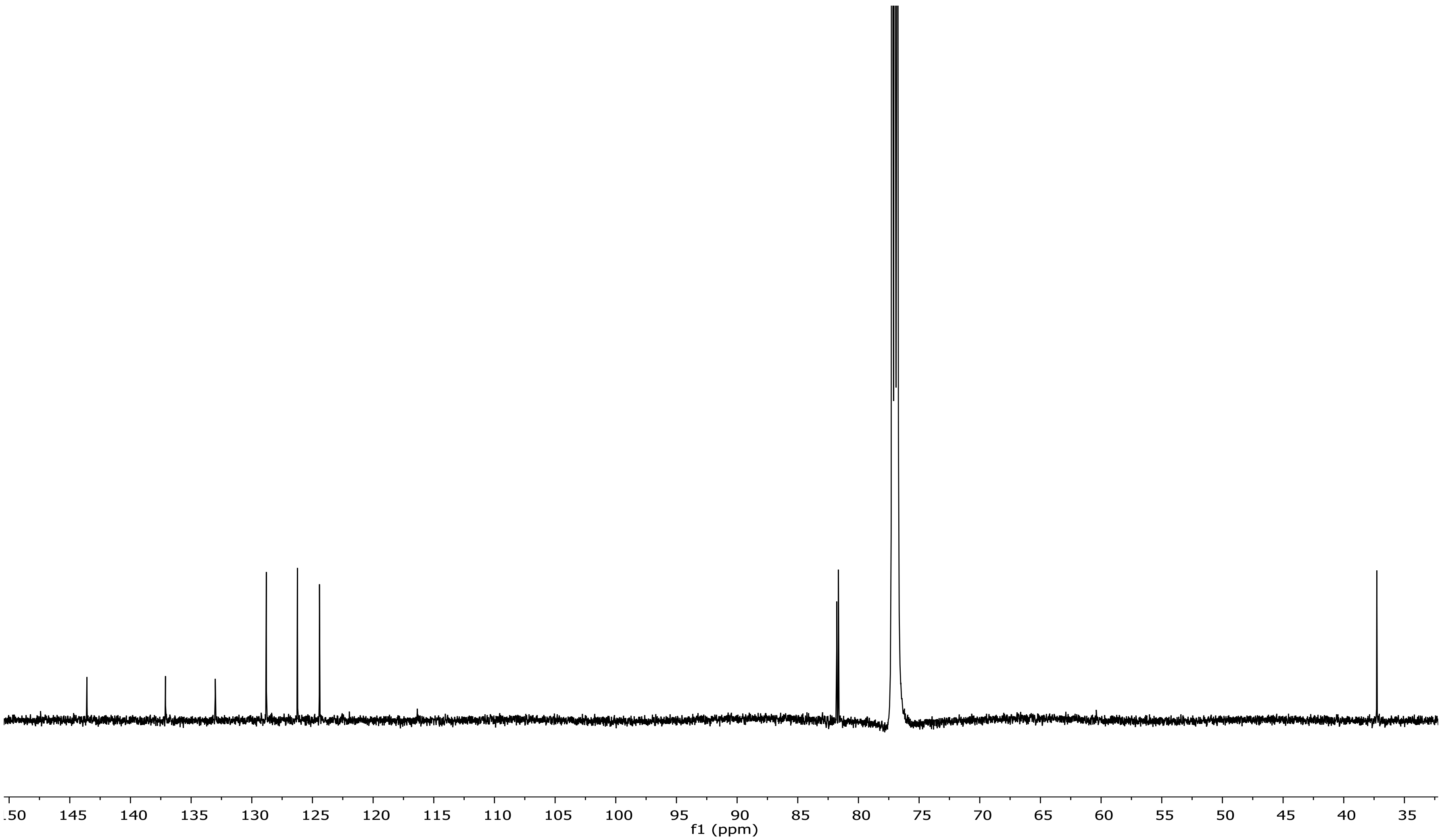

Figure S2. ^{13}C NMR spectrum of *anti*-(+)-6-chloroindan-1,2-diol (*anti*-(+)-**7**) in $CDCl_3$

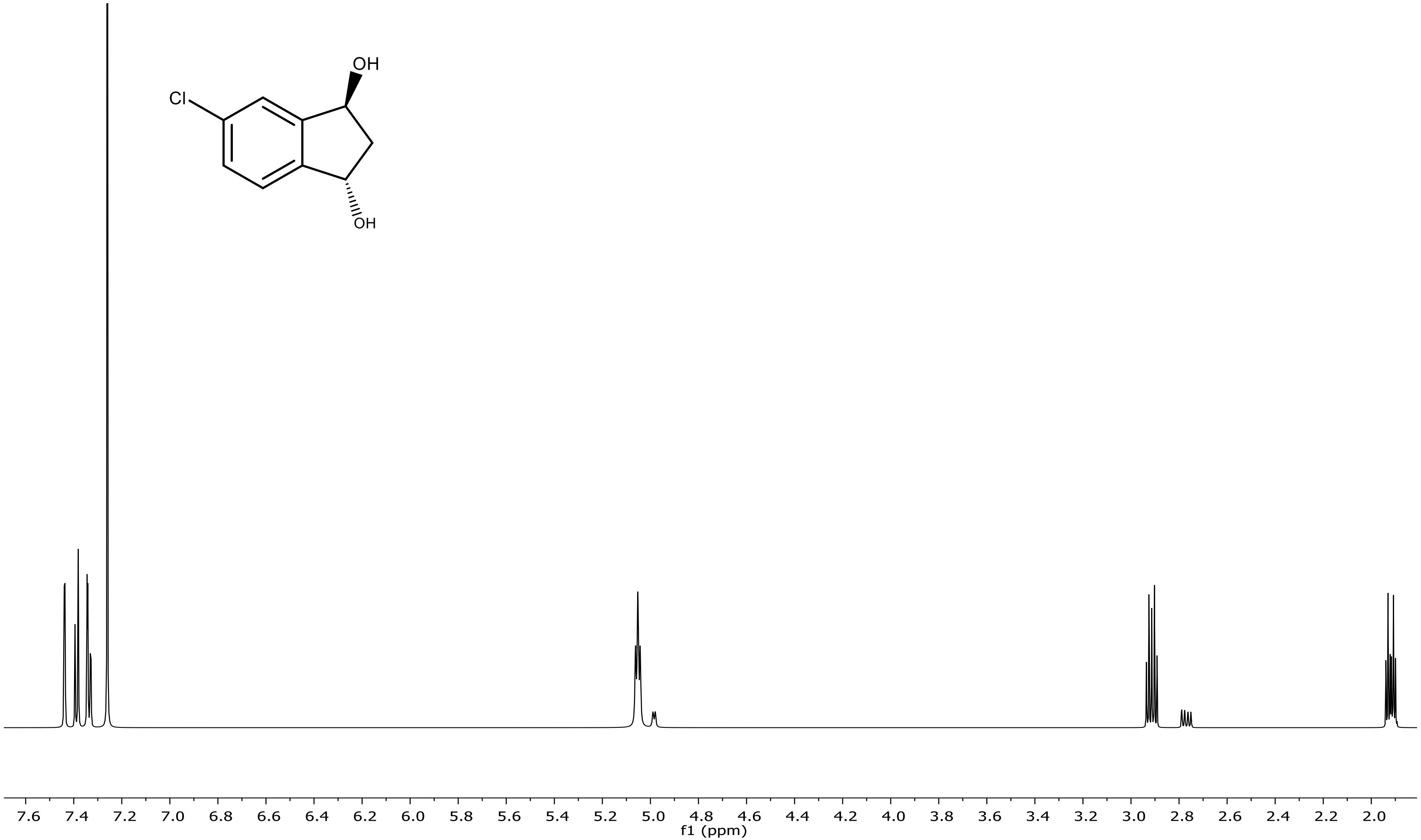

Figure S3. ^{1}H NMR spectrum of *anti*-(+)-5-chloroindan-1,3-diol (*anti*-(+)-**8**) in $CDCl_3$

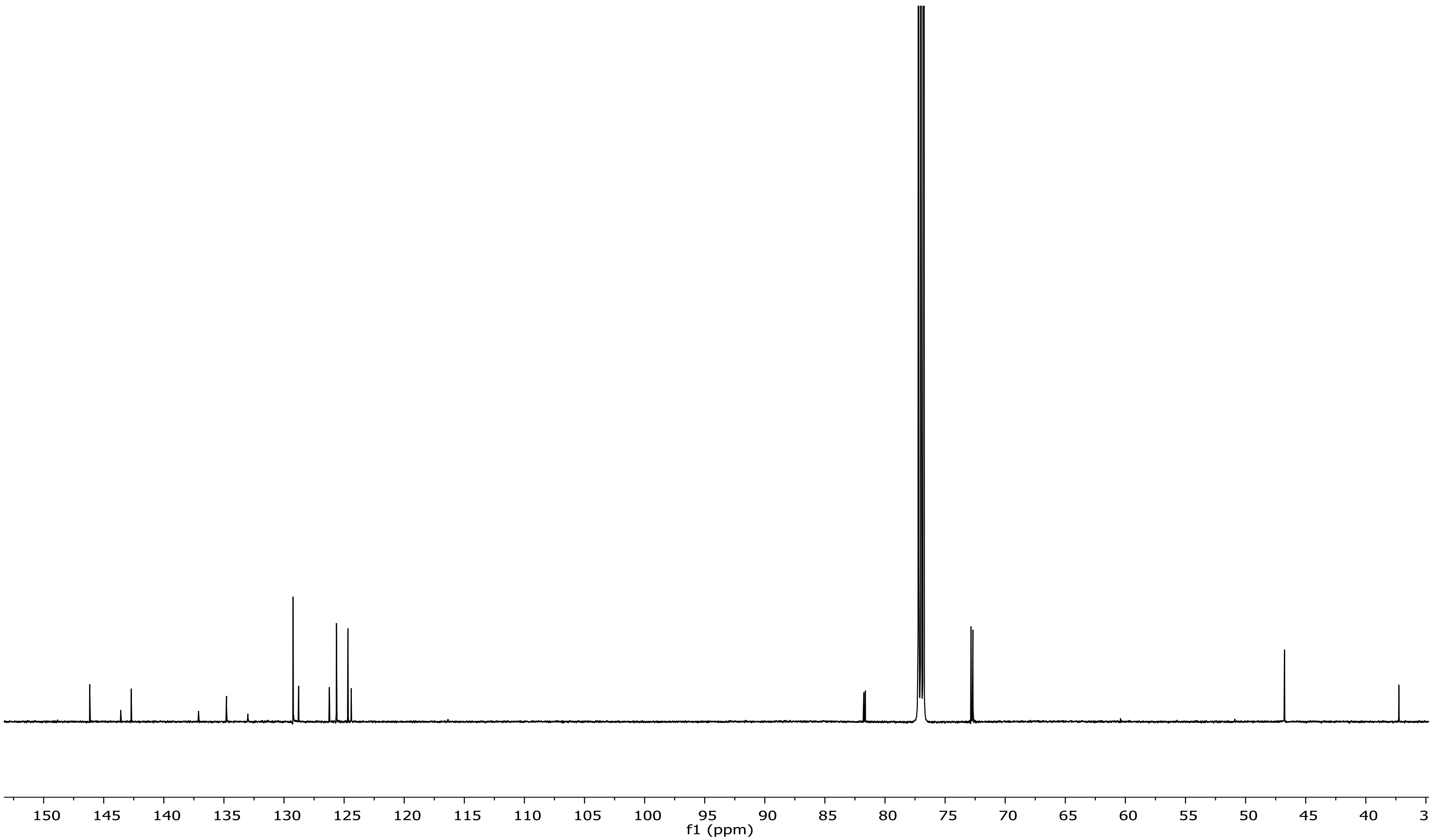

Figure S4. ^{13}C NMR spectrum of of *anti*-(+)-5-chloroindan-1,3-diol (*anti*-(+)-**8**) in $CDCl_3$

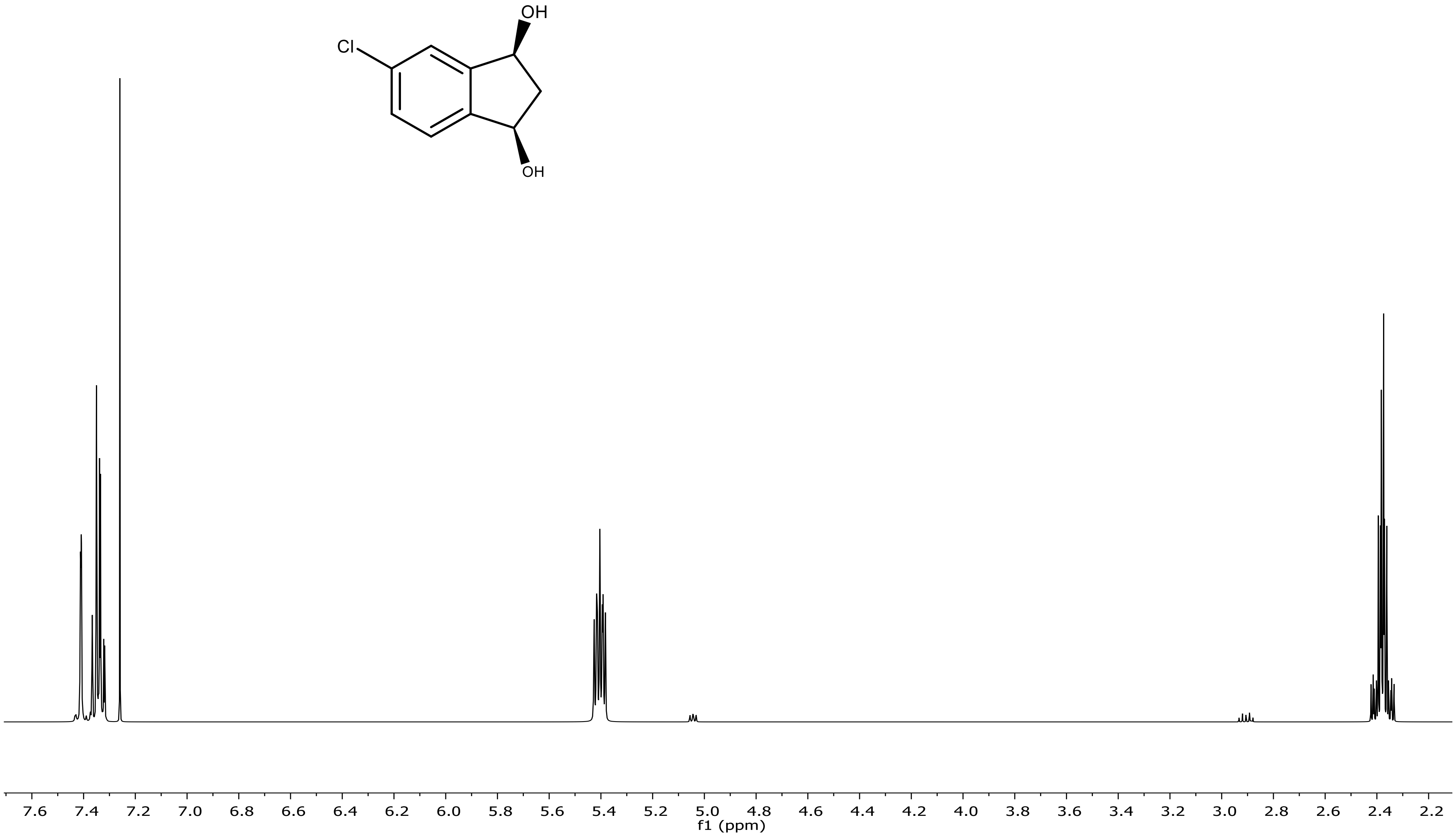

Figure S5. ^{1}H NMR spectrum of *syn*-(-)-5-chloroindan-1,3-diol (*syn*-(-)-**8**) in $CDCl_3$

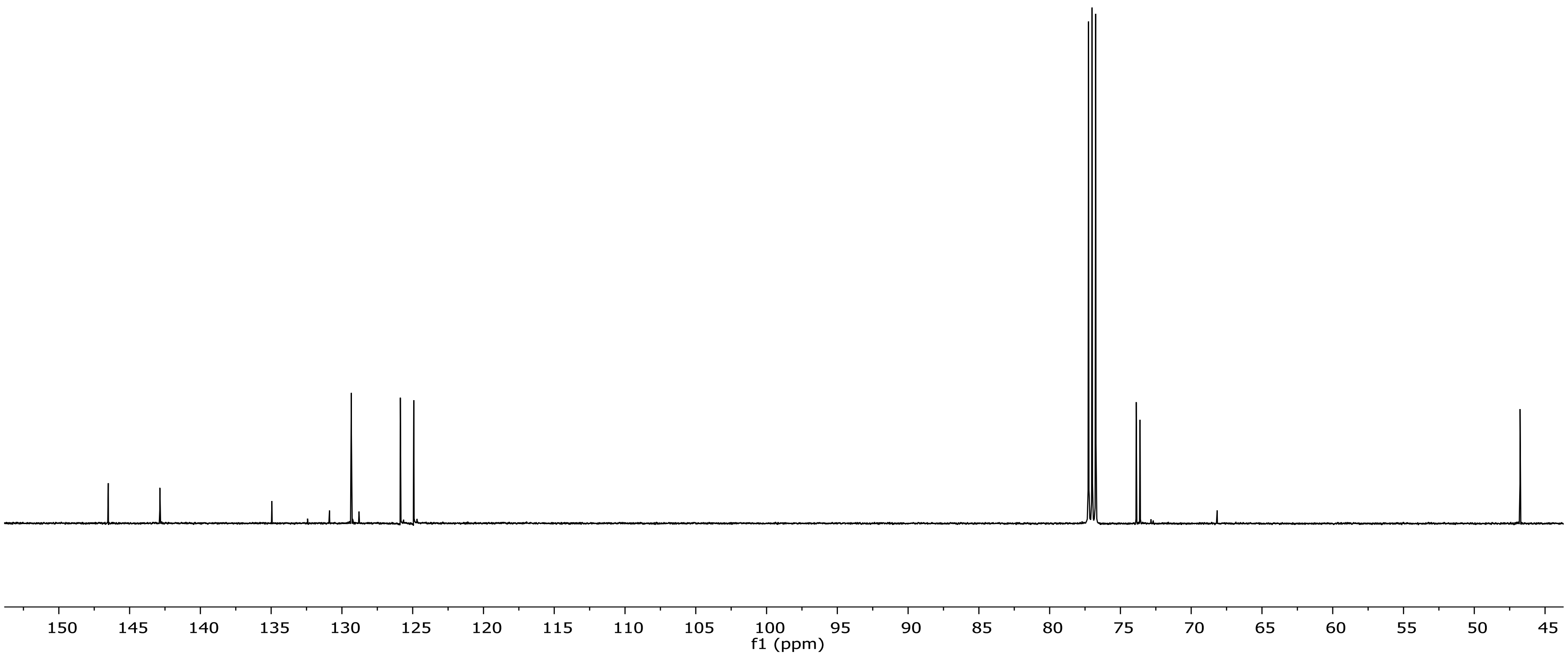

Figure S6. ^{13}C NMR spectrum of *syn*-(-)-5-chloroindan-1,3-diol (*syn*-(-)-**8**) in $CDCl_3$

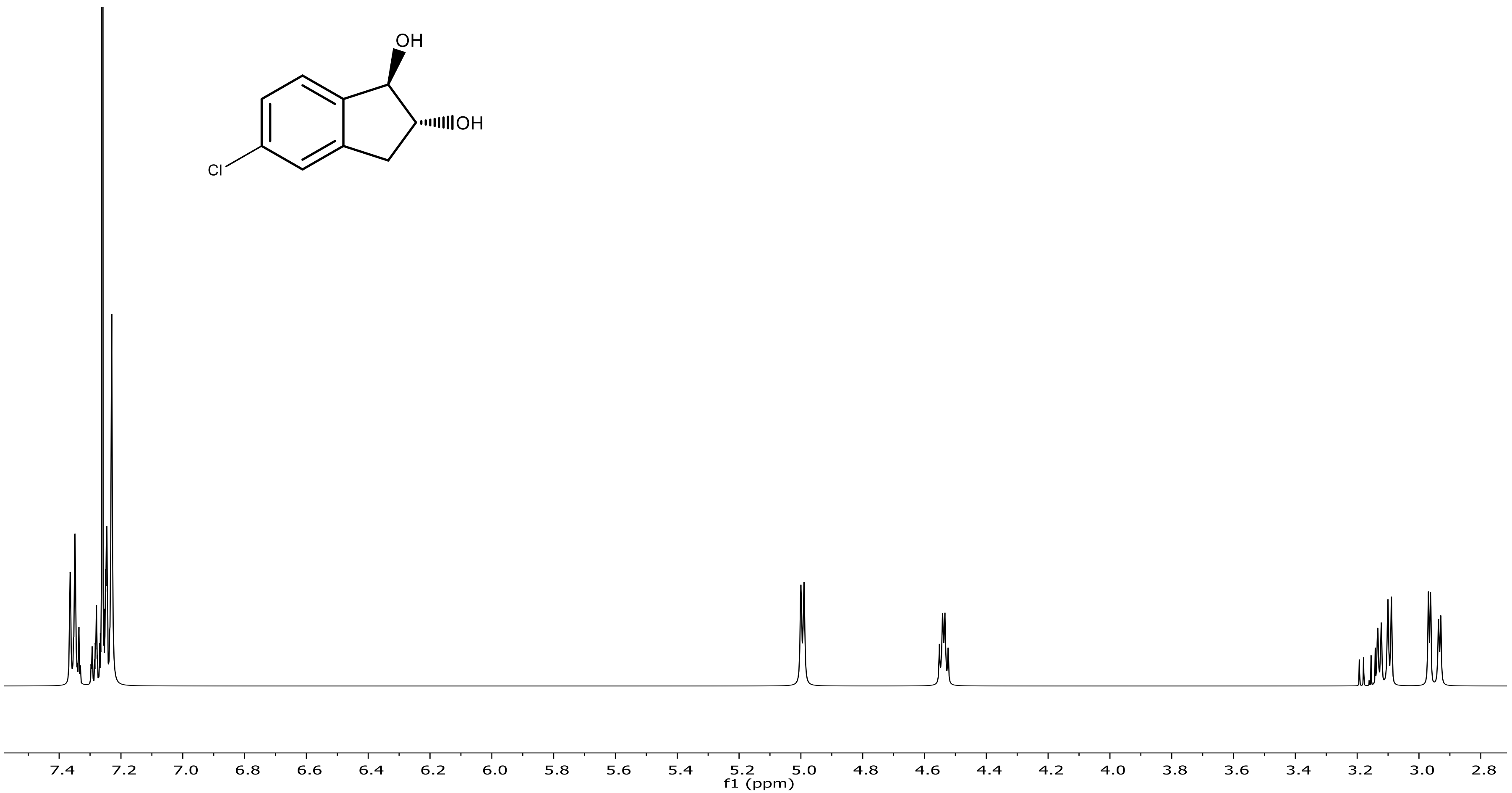

Figure S7. ^{1}H NMR spectrum of *anti*-(+)-5-chloroindan-1,2-diol (*anti*-(+)-**9**) in $CDCl_3$

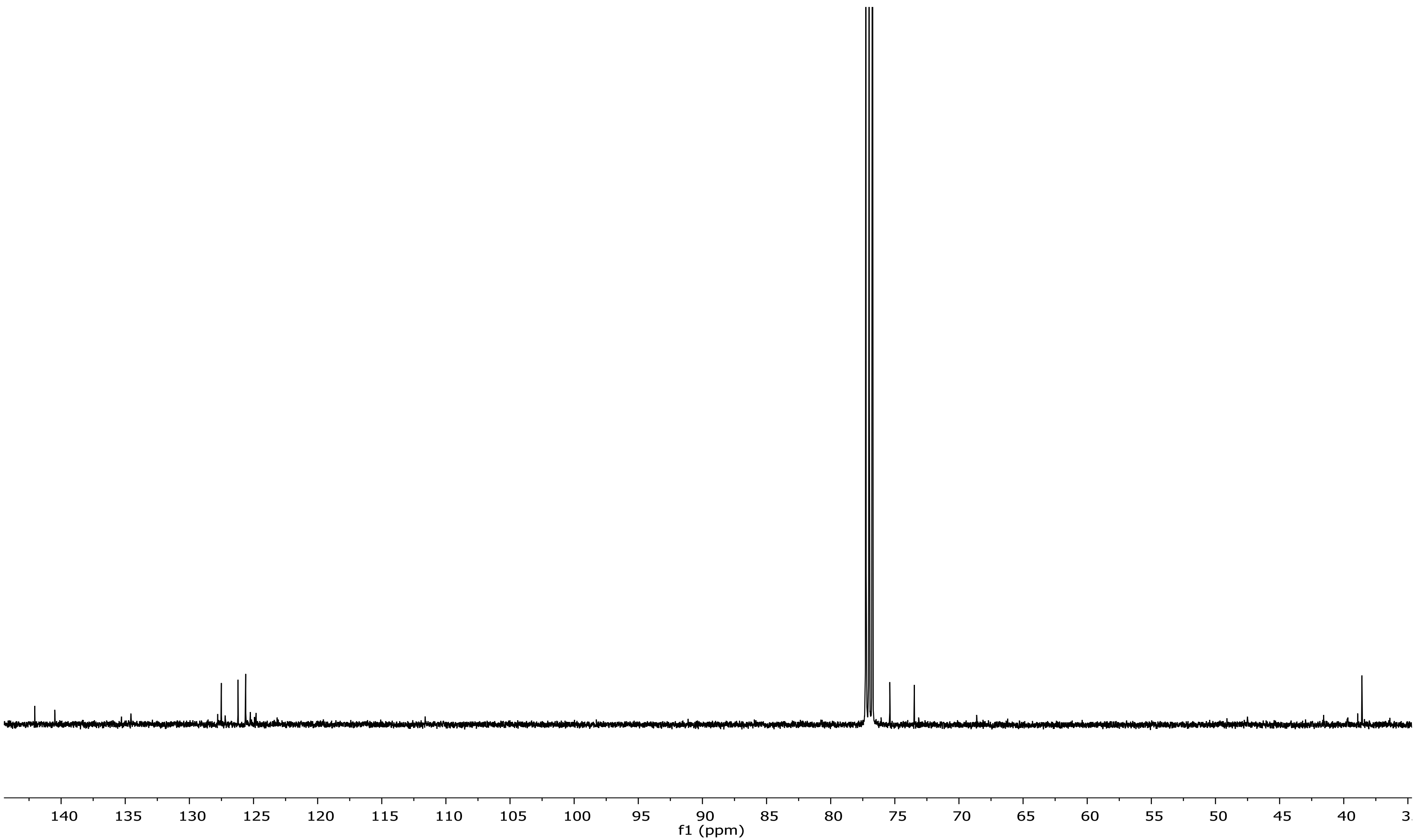

Figure S8. ^{13}C NMR spectrum of *anti*-(+)-5-chloroindan-1,2-diol (*anti*-(+)-**9**) in $CDCl_3$

Figure S9. Tables of fungal growth inhibition for *Botrytis cinerea* UCA992

**6-chloroindanone (3)**				
Hours	**24**	**48**	**72**	**96**
**1**	98,22%	96,00%	79,42%	71,98%
**2**	97,70%	95,42%	79,85%	73,59%
**3**	99,69%	96,91%	79,74%	71,72%
Mean	98,54%	96,11%	79,67%	72,43%
**σ^2**	7,1016E-05	3,7607E-05	3,3267E-06	6,8407E-05
AD*	0,00768889	0,00533333	0,00166667	0,00773333
SD**	0,00842707	0,00613243	0,00182392	0,00827083

σ^2 variance; * average deviation; **standard deviation.

**6-chloroindanol (1)**				
Hours	**24**	**48**	**72**	**96**
**1**	79,96%	65,57%	58,68%	51,16%
**2**	81,78%	65,14%	57,89%	47,59%
**3**	82,90%	66,30%	56,89%	50,05%
**Me**an	81,55%	65,67%	57,82%	49,60%
**σ^2**	0,00014678	2,2927E-05	5,3647E-05	0,00022254
AD*	0,01057778	0,0042	0,0062	0,0134
SD**	0,01211537	0,00478818	0,00732439	0,01491777

σ^2 variance; * average deviation; **standard deviation.

**(*R*)-(-)-6-chloroindanol ((*R*)-1)**				
Hours	**24**	**48**	**72**	**96**
**1**	84,12%	55,05%	53,10%	48,59%
**2**	68,25%	60,67%	50,76%	45,79%
**3**	68,25%	57,86%	56,62%	48,59%
Mean	73,54%	57,86%	53,49%	47,66%
**σ^2**	0,00559682	0,00052641	0,00058006	0,00017422
AD*	0,07053333	0,01873333	0,02084444	0,01244444
SD**	0,0748119	0,02294355	0,02408448	0,01319933

σ^2 variance; * average deviation; **standard deviation.

**(*S*)-(+)-6-chloroindanol ((*S*)-1)**				
Hours	**24**	**48**	**72**	**96**
**1**	76,19%	80,33%	67,17%	56,07%
**2**	84,12%	80,33%	68,34%	60,74%
**3**	84,12%	71,91%	66,00%	57,97%
Mean	81,48%	77,52%	67,17%	58,26%
**σ^2**	0,00139744	0,00157548	9,126E-05	0,00036769
AD*	0,03524444	0,03742222	0,0078	0,01653333
SD**	0,03738238	0,03969226	0,00955301	0,01917516

σ^2 variance; * average deviation; **standard deviation.

**5-chloroindanone (4)**				
Hours	**24**	**48**	**72**	**96**
**1**	75,86%	56,75%	53,38%	49,68%
**2**	71,87%	56,57%	54,23%	50,63%
**3**	57,14%	50,00%	53,44%	35,94%
Mean	68,29%	54,44%	53,68%	45,42%
σ^2	0,00648146	0,00098622	1,5002E-05	0,0045054
AD*	0,07433333	17,79585	0,00364444	0,06317778
SD**	0,08050752	0,03140414	0,00387327	0,06712229

σ^2 variance; * average deviation; **standard deviation.

5-chloroindanol (2)

Hours	24	48	72	96
**1**	58,62%	64,86%	57,62%	55,97%
**2**	71,87%	61,84%	61,01%	59,49%
**3**	75,86%	50,00%	47,41%	45,09%
Mean	68,78%	58,90%	55,35%	53,52%
σ^2	0,00543	0,00411251	0,00334107	0,00375694
AD*	0,06775556	0,05933333	0,05291111	0,05617778
SD**	0,07368855	0,06412883	0,05780198	0,0612939

σ^2 variance; * average deviation; **standard deviation.

		**(*R*)-(-)-5-chloroindanol ((*R*)-2)**		
Hours	**24**	**48**	**72**	**96**
**1**	82,75%	66,21%	62,71%	66,66%
**2**	75,00%	76,31%	67,79%	65,18%
**3**	57,14%	60,52%	67,24%	64,05%
Mean	71,63%	67,68%	65,91%	65,30%
σ^2	0,01149905	0,00426345	0,00051811	0,00011422
AD*	0,0966	0,05753333	0,02135556	0,00908889
SD**	0,10723361	0,06529507	0,02276201	0,01068717

σ^2 variance; * average deviation; **standard deviation.

		**(*S*)-(+)-5-chloroindanol ((*S*)-2)**		
Hours	**24**	**48**	**72**	**96**
**1**	100,00%	100,00%	91,30%	90,40%
**2**	100,00%	100,00%	100,00%	82,85%
**3**	100,00%	100,00%	96,00%	97,14%
Mean	100,00%	100,00%	95,77%	90,13%
σ^2	0	0	0,00126422	0,00340705
AD*	0	0	0,02977778	0,04853333
SD**	0	0	0,0355559	0,05836991

σ^2 variance; * average deviation; **standard deviation.

		**Dichlofluanid**		
Hours	**24**	**48**	**72**	**96**
**1**	100,00%	100,00%	100,00%	96,54%
**2**	100,00%	100,00%	100,00%	95,33%
**3**	100,00%	100,00%	100,00%	97,87%
Mean	100,00%	100,00%	100,00%	96,58%
σ^2	0	0	0	0,00010761
AD*	0	0	0	0,0086
SD**	0	0	0	0,01037336

σ^2 variance; * average deviation; **standard deviation.
